# Giant cardiac hemangioma in the right atrium: an asymptomatic surgical case

**DOI:** 10.1186/s44215-023-00060-3

**Published:** 2023-07-30

**Authors:** Yuki Kondo, Toru Yasutsune, Yuichiro Kado, Yuki Jinzai, Tomoya Takigawa, Takehiro Kishigami, Yuna Inaba, Yosuke Nishimura

**Affiliations:** 1 Department of Cardiovascular Surgery, School of Medicine, University of Occupational and Environmental Health, 1-1, Iseigaoka, Yahata-Nishi-Ku, Kitakyushu, Fukuoka 807-8556 Japan; 2 Department of Pathology, School of Medicine, University of Occupational and Environmental Health, Kitakyushu, Japan

**Keywords:** Cardiac tumor, Hemangioma, Right atrium

## Abstract

**Background:**

Cardiac hemangiomas are rare, accounting for only 5% of benign cardiac tumors. In the past, there have been few reports of giant cardiac hemangiomas that were > 100 mm in size but were asymptomatic.

**Case presentation:**

A 44-year-old woman presented with a large asymptomatic intracardiac mass that was accidentally detected on echocardiography. The tumor was surgically resected. During surgery, a sharply margined tumor was located in the right atrium; the tumor was histopathologically diagnosed as a cavernous hemangioma. The patient was discharged uneventfully on the 18th postoperative day. No signs of recurrence were observed at 1 year postoperatively.

**Conclusions:**

We report on a surgical case of an asymptomatic giant cardiac hemangioma 115 mm × 92 mm in size, as measured by echocardiography. It is difficult to diagnose cardiac tumors before surgery based on symptoms and imaging. Surgical resection is the most reliable treatment because of its accurate diagnosis and favorable prognosis.

## Background

The incidence of cardiac hemangiomas is reported to be only 5% among benign cardiac tumors [1]. A previous review reported that the average size of cardiac hemangiomas is approximately 50 mm [2]. There have been few previous reports of giant cardiac hemangiomas. Herein, we report an asymptomatic surgical case of a giant cardiac hemangioma > 100 mm in size in the right atrium (RA).

## Case presentation

**Fig. 1:**
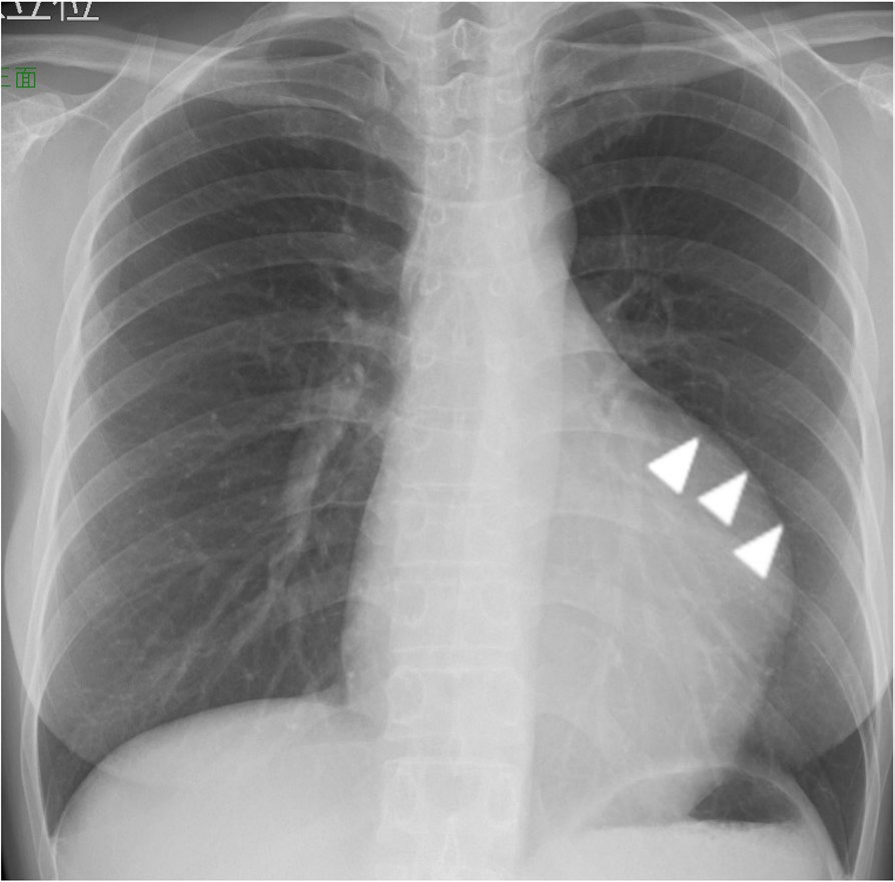
Chest X-ray showing protrusion of the left upper cardiac margin (white arrowhead)

**Fig. 2:**
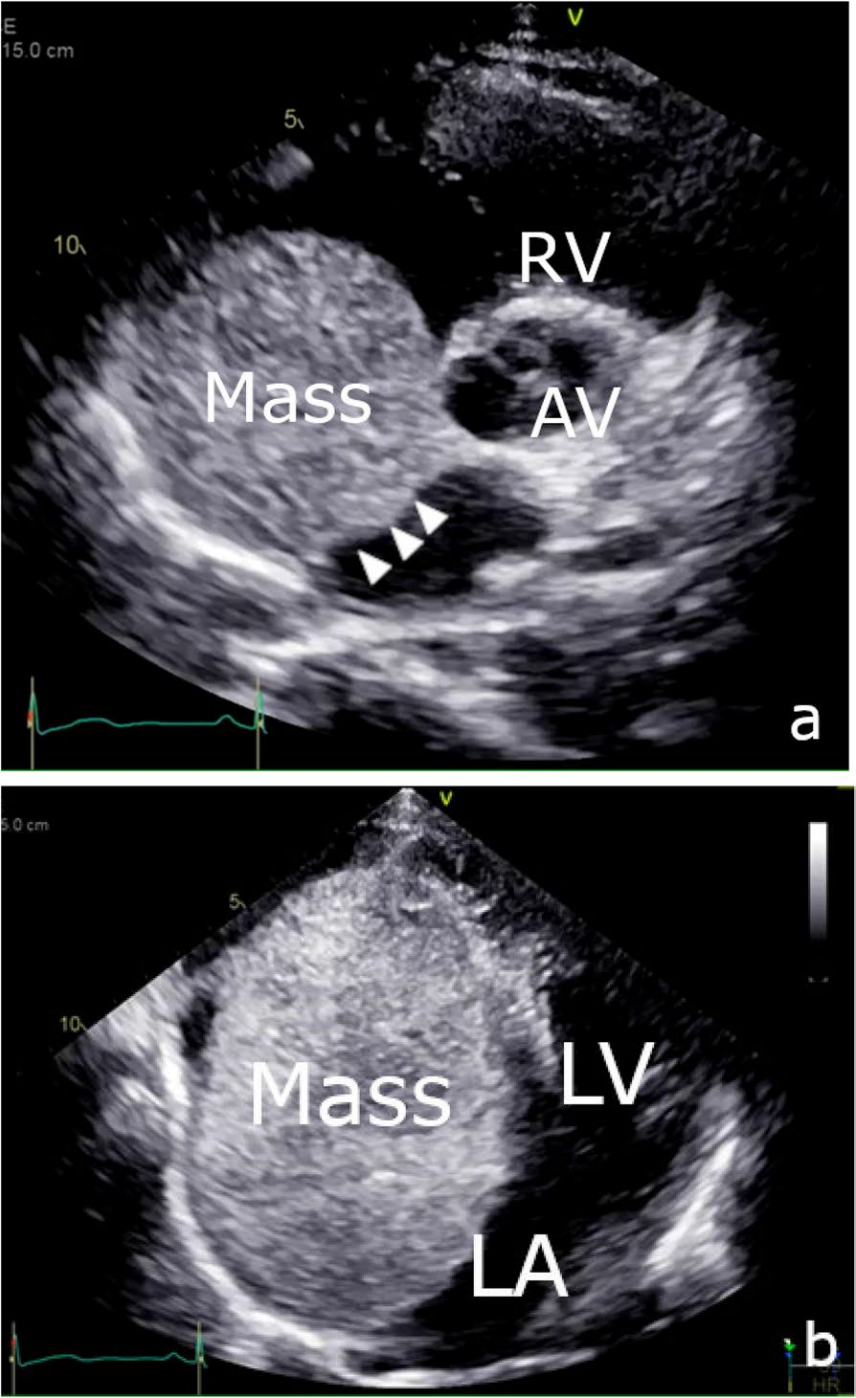
Preoperative transthoracic echocardiography. A short-axis view of aortic valve level (**a**) showing the mass with a smooth surface attaching the septal wall (white arrowhead) and a four-chamber view (**b**) showing the mass compressing both heart ventricles. The mass measures 115 × 92 mm in size without ultrasonic signals of blood flow. AV, aortic valve; LA, left atrium; LV, left ventricle; RA, right atrium; RV, right ventricle

**Fig. 3:**
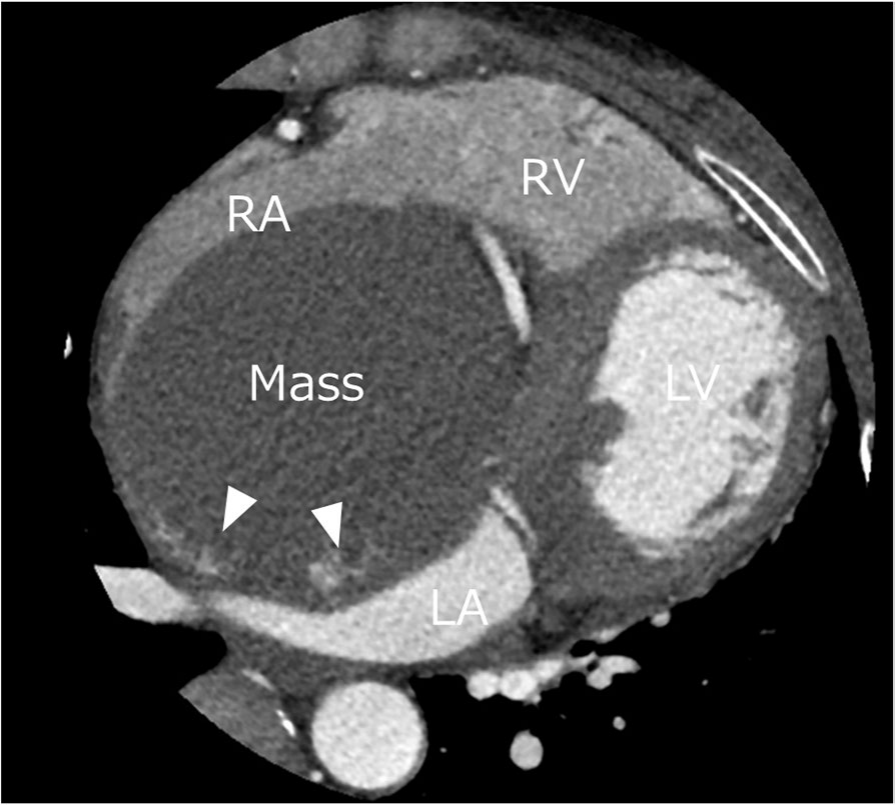
Contrast-enhanced computed tomography. The right atrial chamber is occupied by the mass having partial contrast enhancement, which suggests the possibility of feeding arteries. LA, left atrium; LV, left ventricle; RA, right atrium; RV, right ventricle

**Fig. 4:**
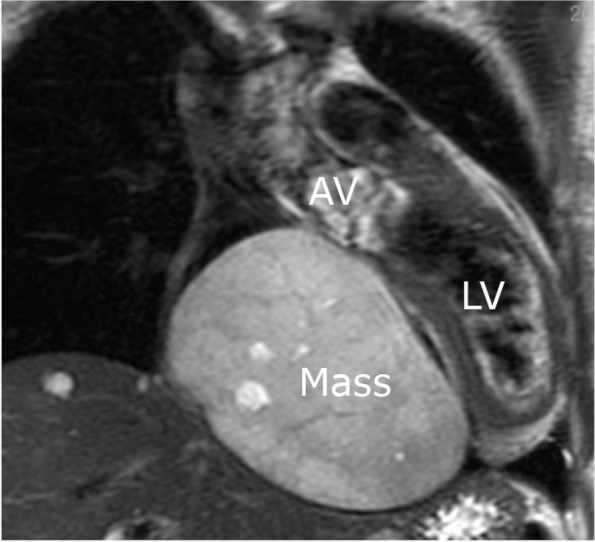
A magnetic resonance image on T2 (coronal plane)-weighted image showing the tumor has a high signal intensity. The mass is compressing LV toward the left side, with no significant evidence of infiltration. AV, aortic valve; LV, left ventricle

A 44-year-old Japanese woman underwent chest radiography during an annual medical checkup, which revealed cardiomegaly. The patient was referred to our hospital for further evaluation. She had a medical history of a benign thyroid tumor and left maxillary sinusitis. The blood test showed no significant findings (Table 1). The chest X-ray indicated 55% of cardiothoracic ratio and protrusion of the left upper cardiac margin (Fig. 1). Transthoracic echocardiography revealed a mass (115 × 92 mm) with a smooth surface attached to the septal wall of the RA (Fig. 2a). The mass compressed the ventricles of the heart (Fig. 2b). No significant ultrasonic signals of blood flow were observed in the mass. Computed tomography with contrast agents showed a sharply margined mass having partial contrast enhancement in the RA, which meant this mass might have feeding arteries (Fig. 3). Magnetic resonance imaging showed a sharply margined mass (Fig. 4) with no apparent evidence of malignancy such as infiltration or metastasis although it could not be denied that the tumor was malignant due to its size. The mass had a high signal intensity on T2-weighted images and diffuse restriction on diffusion-weighted images.

**Table 1:** Blood test findings on admission

Complete blood count	Coagulation	Biochemistry	
White cell count: 5.8 × 10^3^/µl Red cell count: 3.79 × 10^6^/µl Hemoglobin: 10.4 g/dl Hematocrit: 34.8% Mean cell volume: 83.9 µm^3^ Platelet count: 286 × 10^3^/µl	Prothrombin time: 11.9 s International normalized ratio: 1.02 Activated partial thromboplastin time: 29.9 s	Total protein: 6.6 g/dl Albumin: 4.0 g/dl Total bilirubin: 0.8 mg/dl Aspartate aminotransferase: 20 U/l Alanine aminotransferase: 21 U/l Alkaline phosphatase: 48 U/l Gamma-glutamyl transferase: 17 U/l Total cholesterol: 175 mg/dl Triacylglycerol: 42 mg/dl High-density lipoprotein: 88 mg/dl Low-density lipoprotein: 91 mg/dl Blood urea nitrogen: 9 mg/dl Creatinine: 0.5 mg/dl	Sodium: 138 mmol/l Potassium: 4.2 mmol/l Chloride: 107 mmol/l Calcium: 8.9 mg/dl Creatinine kinase: 190 U/l C-reactive protein: 0.01 mg/dl NT-pro brain natriuretic peptides: 131 pg/ml Hemoglobin A1c: 5.6% Thyroid-stimulating hormone: 0.20 IU/ml Free T3 (triiodothyronine): 2.87 pg/ml Free T4 (thyroxine): 1.02 ng/dl Carcinoembryonic antigen: 0.7 ng/ml Carbohydrate antigen 19–9: 5.0 U/ml

We found trivial anemia and no other significant findings including the hormone markers and the tumor makers

**Fig. 5:**
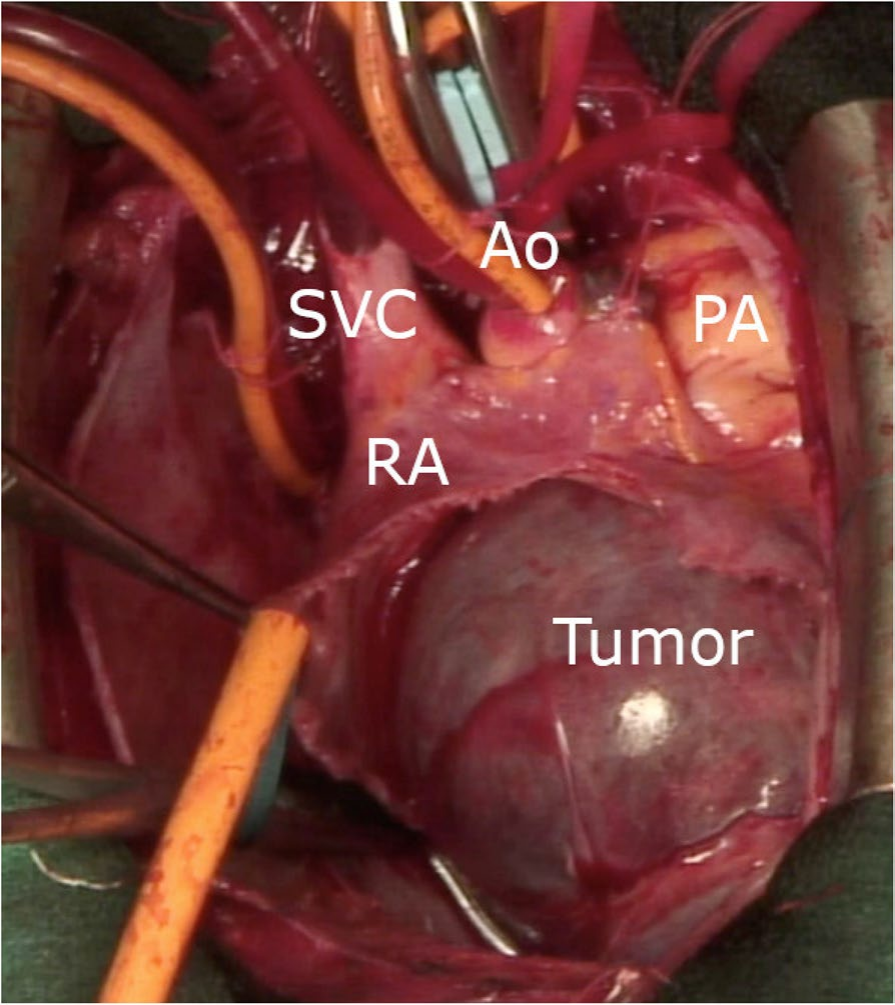
Operative findings. A sharply margined tumor with a smooth surface in the RA and occupying most of the RA cavity. The tumor arose from the atrial septum between the oval fossa and coronary sinus. Ao, aorta; PA, pulmonary artery; SVC, superior vena cava; RA, right atrium

**Fig. 6:**
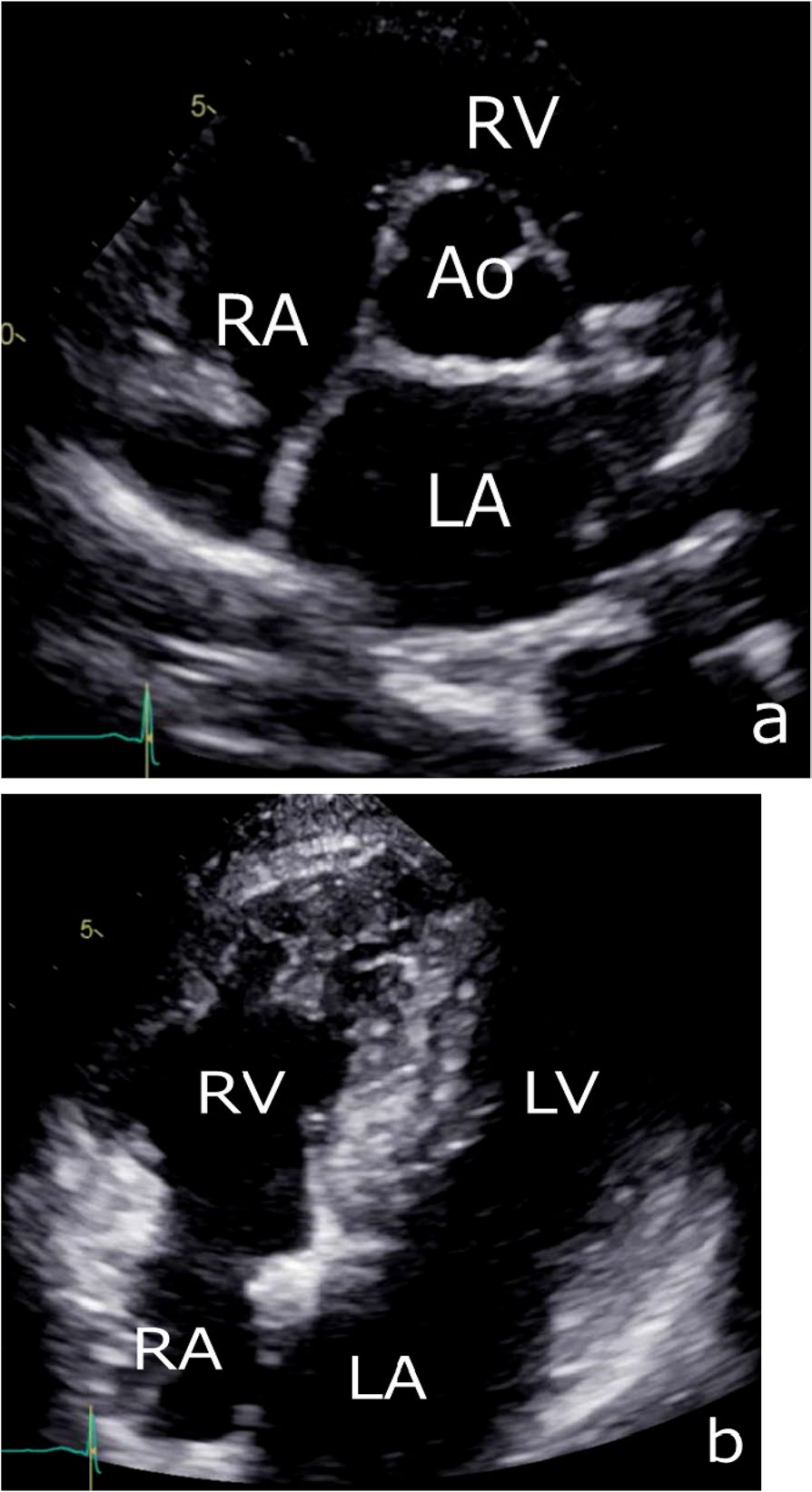
Postoperative transthoracic echocardiography showed RA volume reduction without transarterial shunt flow or residual mass. AV, aortic valve; LA, left atrium; LV, left ventricle; RA, right atrium; RV, right ventricle

**Fig. 7:**
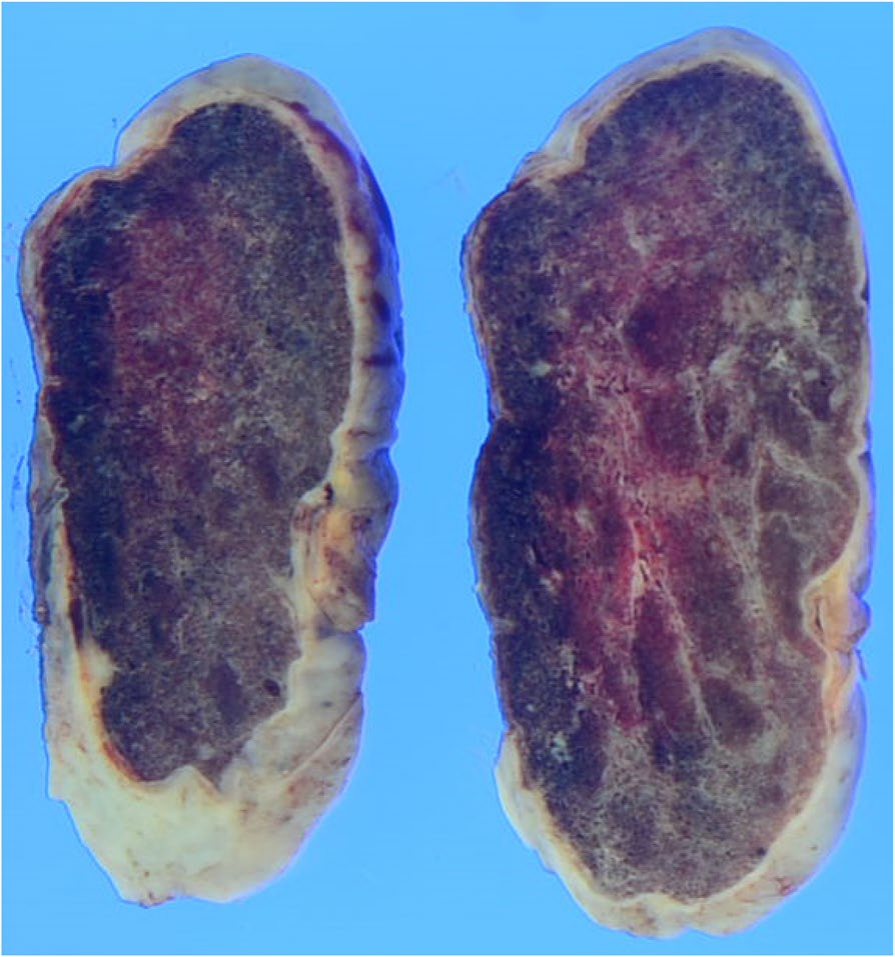
The cross-section of the tumor revealing clusters of small vessels

**Fig. 8:**
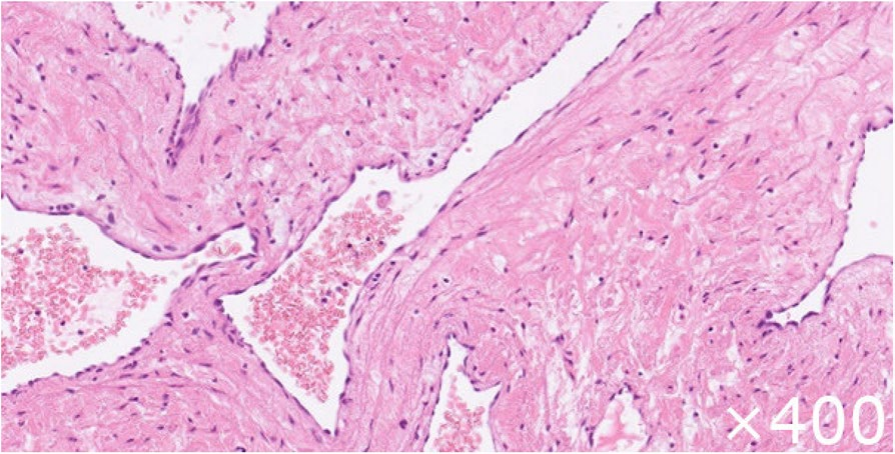
Histopathological findings with hematoxylin and eosin staining. Multiple dilated vascular channels with endothelial cell lining are observed throughout

We decided to perform surgery to prevent severe complications such as hemodynamic insufficiency or tumor embolism and thereafter made a precise diagnosis by pathological examination. Surgery was performed using the median sternotomy approach under general anesthesia. Cardiopulmonary bypass was established with bicaval venous drainage and systemic arterial perfusion via the ascending aorta. Access to the inferior vena cava (IVC) was achieved with peripheral cannulation via the right common femoral vein as it can be difficult to safely perform direct cannulation of the IVC. The aorta was cross-clamped, and cardiac arrest was induced with antegrade cold blood cardioplegia. A longitudinal incision in the RA was made parallel to the atrioventricular groove (Fig. 5). The tumor was found to fill the right atrial space and was attached to the atrial septum between the oval fossa and the coronary sinus. Tumor extirpation was successfully performed. Reconstruction of the right-sided wall defect of the atrial septum was performed using an autologous pericardial patch. Postoperative transthoracic echocardiography showed no transarterial shunt flow or residual mass (Fig. 6). A cross-section of the tumor revealed a cavernous appearance (Fig. 7). Histopathologically, the tumor was composed of multiple dilated vascular channels with endothelial cell lining and was diagnosed as a cavernous-type cardiac hemangioma (Fig. 8). The patient had an uneventful postoperative course and was discharged on the 18th postoperative day. No signs or evidence of recurrence was observed 1 year after the operation.

## Discussion

Cardiac tumors are classified as primary and metastatic tumors, with a reported ratio of 1:30 [3]. Primary cardiac tumors are rare, with an incidence of 0.002 to 0.3% at autopsy [1]. Benign tumors make up 75% of cardiac primary tumors, and hemangiomas account for only 5% of benign cardiac tumors [1]. The incidence rate of cardiac hemangiomas is slightly higher in women than in men [2].

According to previous reports, the locations of cardiac hemangiomas may vary. Miao and colleagues [2] reported that 35.8% of atrial cardiac hemangiomas are located in the left atrium, 63.7% in the RA, and 1.5% in the biatrium. Kojima et al. [4] reported that 36% of cardiac hemangiomas were located in the right ventricle, 34% in the left ventricle, 23% in the RA, 11% in the atrial septum, 11% in the ventricular septum, and 7% in the left atrium. Histopathologically, cardiac hemangiomas are categorized as cavernous, capillary, and atriovenous types, with the cavernous type being the most common, accounting for 58.5% of cardiac hemangiomas [2].

The average size of cardiac hemangiomas is 52.3 mm [2]. To the best of our knowledge, the largest hemangioma measured 280 × 35 mm, as reported by Rivera and colleagues [5], and the patient developed syncope. Most cardiac tumor cases are asymptomatic, but symptoms vary depending on the age of the patient and the location and size of the mass. Cardiac tumors can lead to life-threatening embolisms, incarceration, fatal arrhythmia, and hemodynamic collapse, necessitating early intervention. Because hemangiomas may occur simultaneously in multiple organs, including the heart, liver, skin, pleura, and lungs [2], patients should undergo systemic examinations.

We searched PubMed to identify relevant case reports using the following terms: “giant cardiac tumor” or “large cardiac tumor.” Patients with tumors located outside the heart were excluded. Fourteen articles [5–18] with a tumor size of > 100 mm were identified (Table 2). Eight of the 14 cases were myxomas, and the other six were hemangiomas. It has been suggested that giant cardiac tumors are more likely to be hemangiomas. Of the 14 patients, four were asymptomatic. The tumor size in our asymptomatic patient was 115 × 92 mm. The tumor compressed both ventricles but did not cause hemodynamic insufficiencies such as heart failure, intracavity obstruction, and valvular dysfunction. We believe that the locations of cardiac tumors are associated with displayed symptoms. Table 2 shows that the giant cardiac tumors predominantly originated from the atrial septal walls, which suggests that the tumors originating from the atrial septum were not easily accessible, and are less able to interfere with hemodynamics.

**Table 2:** Fourteen cases of benign giant cardiac tumors with a size of more than 100 mm. Eight of fourteen cases with myxomas and the other six with hemangiomas having tumors predominantly originating from the atrial septal wall

First author	Year	Age	Sex	Types	Size (mm)	Chamber	Origination of the tumor	Symptoms
Pigato JB [6]	1998	74	F	Hemangioma	100	LA	Posterior wall of the LA	Shortness of breath
Jimenez-Navaro MF [7]	2001	48	M	Myxoma	120 × 50	RA	NA	Hepatic dysfunction
Lamparter S [8]	2004	70	F	Myxoma	100 × 30	LA	Posterior wall of the LA	Asymptomatic
Zanati SG [9]	2008	30	M	Hemangioma	130 × 120	RA, RV	NA	Cough, chest pain
Panagiotou M [10]	2008	58	M	Myxoma	120 × 100	LA	Septum	Asymptomatic
Mongal LS [11]	2009	42	F	Hemangioma	110 × 65	LA	Septum	Asymptomatic
Husian Z [12]	2011	42	F	Hemangioma	110 × 65	RA	Septum	Shortness of breath
Yilmaz F [13]	2012	78	F	Myxoma	105 × 45	LA, LV	NA	Syncope
Sato T [14]	2012	75	F	Myxoma	125 × 75 × 20	RA	Septum	Fatigue, cough, hepatic dysfunction
Nina V JS [15]	2012	45	W	Myxoma	100 × 60 × 80	RA	Atrial superior vena cava junction of the RA	Palpitation, dyspnea
Perez Rivera CJ [5]	2019	48	F	Hemangioma	280 × 35	RA	NA	Syncope
Dibrtioiu F [16]	2020	52	F	Hemangioma	110	RA	NA	Asymptomatic
Al-Zamkan BK [17]	2020	54	F	Myxoma	100 × 80 × 60	RA	Septum	Dyspnea, palpitation
Fan C [18]	2021	55	M	Myxoma	109 × 44, 24 × 18	RA, LA	Septum	Shortness of breath

*F* female, *M* male, *RA* right atrium, *LA* left atrium, *NA* not available

There may be alternative treatments for cardiac hemangiomas; however, surgery is the most reliable method for diagnosis. Surgical resection of cardiac tumors is generally performed, and reconstruction of the wall deficit may be performed, if necessary. Hoffmeier and colleagues [19] reported that the 5-year survival rate of benign cardiac tumors after surgical treatment was 83%, and the 10-year survival rate was 75%. A few reports have shown that cardiac hemangiomas recur and transform into angiosarcomas [20]. Therefore, in addition to complete surgical resection, diagnosis of whether the tumor is benign or malignant is particularly important for patient prognosis. In our case, we found no evidence of recurrence, although we only tracked the patient for 1 year after surgery. Therefore, further observation of this patient is warranted.

## Conclusions

Here, we report a case of an asymptomatic giant cardiac hemangioma originating from the right atrial septal wall that was surgically resected. Although some benign cardiac tumors grow massively, they do not always manifest with specific symptoms and are not easily diagnosed preoperatively. Surgical resection is the most effective therapy for precise diagnosis and prevention of detrimental complications.

## Data Availability

All data generated or analyzed during this study are included in this published article.

## Abbreviations

AV: Aortic valve
F: Female
LA: Left atrium
LV: Left ventricle
M: Male
NA: Unavailable
PA: Pulmonary artery
RA: Right atrium
RV: Right ventricle
SVC: Superior vena cava
